# Experimental Investigation on Thermo-Mechanical, Visco-Elastic, and Acoustic Properties of *Hibiscus rosa-sinensis* Plant Fiber-Reinforced Polymer Composites

**DOI:** 10.3390/polym18101189

**Published:** 2026-05-13

**Authors:** M. Ramesh, M. Tamil Selvan, L. Rajeshkumar, P. Ramya

**Affiliations:** 1 Department of Mechanical Engineering, Mahendra Engineering College, Namakkal 637503, Tamil Nadu, India; 2 Department of Mechanical Engineering, Sri Eshwar College of Engineering, Coimbatore 641202, Tamil Nadu, India; tamilmmech@gmail.com; 3 CoE–Advanced Materials Synthesis, Department of Mechanical Engineering, Alliance School of Applied Engineering, Alliance University, Bengaluru 562106, Karnataka, India; lrkln27@gmail.com; 4 Department of Computer Science and Engineering, Mahendra Engineering College, Namakkal 637503, Tamil Nadu, India; paramasivam.ramya@gmail.com

**Keywords:** *Hibiscus rosa-sinensis* fibers, polymer composites, thermo-mechanical properties, visco-elastic properties, dielectric properties, acoustic properties

## Abstract

Our investigation into *Hibiscus rosa-sinensis* fibers (HRFs) for composite applications involved a multi-step process, primarily fiber extraction through water retting and subsequent surface modification by using sodium hydroxide (NaOH) and trimethoxy methyl silane (TMMS). Through the compression molding technique, untreated HRF-reinforced poly-lactic acid (PLA) composites (UHRFCs), NaOH-treated HRF-reinforced PLA composites (NHRFCs), and TMMS-treated HRF-reinforced PLA composites (THRFCs) were fabricated. The experiments were conducted, and the findings revealed a substantial increase in properties of both NHRFCs and THRFCs compared to UHRFCs. Notably, these enhancements encompassed tensile strength (13.66% and 19.39%), tensile modulus (13.41% and 20.70%), flexural strength (15.98% and 23.17%), flexural modulus (17.13% and 26.58%), impact strength (15.62% and 33.07%), Shore-D hardness (4.19% and 5.00%), storage modulus (9.88% and 13.07%), loss modulus (7.52% and 17.36%), dielectric constant at 6.5 Hz (13.22% and 23.96%), and significant improvements in the acoustic resonance frequency at 1897 Hz (79.50% and 81%). Peak thermal degradation temperatures of these composites are 420.62 ± 3.43 °C, 439.51 ± 3.54 °C, and 469.07 ± 3.11 °C, respectively, and biodegradability results showing accelerated degradation within 30 days. These findings highlight the substantial effectiveness of treatments in enhancing diverse properties, underscoring the potential applicability of these composites in various industrial sectors requiring superior performance and sustainable materials.

## 1. Introduction

In recent times, the quest for eco-friendly and sustainable materials has become a crucial aspect of material research and development. The escalating environmental concerns and the need for minimizing the carbon footprint have motivated scientists to explore alternative materials that can replace traditional synthetic reinforcements in composites [1,2]. Plant fibers have drawn interest among the natural fibers because of their superior mechanical characteristics, the ability to degrade and renewability [3,4,5]. Additionally, these fibers are eco-friendly, as they can absorb CO_2_ while generating oxygen within their lifespan [6]. Furthermore, the decomposition of plant roots and leaves contributes to enhancements in soil fertility, potentially reducing the need for fertilizers [7,8]. To address the escalating utilization of polymers, researchers have initiated the development of bio-inspired green composites [9,10,11]. These environmentally friendly materials are created by reinforcing biodegradable natural fibers into biodegradable polymers [12]. PLA has the ability to degrade and is a bio-based polymer, demonstrating potential as a sustainable substitute for traditional petroleum-based plastics [13,14]. It is biodegradable and can be extracted from agricultural materials like potato, sugar beet, and corn [15,16,17]. It can withstand heavy loads during tensile strength and modulus testing, and it demonstrates durability against impacts. PLA has a glass transition temperature of 55 °C and a melting point of 175 °C, which has gained great interest [14,18,19]. Consequently, researchers are interested in reinforcing PLA with plant fibers to improve its mechanical properties and broadening its prospective applications [20,21,22]. The addition of plant fibers as reinforcement has produced significant enhancement in the mechanical behavior of PLA, characterized by superior strength, stiffness, and impact resistance [23,24,25].

Attempts have been made to enhance the bonding between of the reinforcement and resin in order to overcome the limitations of plant fibers [26,27,28]. Various surface-modification approaches have been investigated, and it was found that the chemical treatments are very effective and useful. These treatments are used to enhance the compatibility of fibers with resin [29]. Chemical treatments bring functional groups onto the fibers’ outer layer, facilitating excellent bonds with the resin and resulting in enhanced load transmission between the two phases [30,31]. As an outcome, chemically treated fibers can enhance the general functionality of the materials. Studies on the mechanical [32,33], thermal [26,34], visco-elastic [34,35], dielectric [24,29], sound absorption [36,37], water absorption [26,38], and biodegradability [39,40] properties of plant fiber-reinforced polymer composites have been conducted by researchers. Several articles have explored the influence of chemical modifications on the outer layer of different plant-based fibers, including jute [41], kenaf [42], sisal [34], bamboo [43], and coir [44], used as reinforcements with polymer matrices.

The mechanical properties of jute, agave cantala, and flax fiber-reinforced PLA composites were investigated at different NaOH concentrations. It was found that NaOH treatment resulted in increased mechanical characteristics, with a 5% concentration providing the highest strength [41]. However, higher NaOH concentrations resulted in a decrease in mechanical properties due to decomposition of the fiber surface [44]. The influence of different NaOH concentrations on the behavior of jute fiber-reinforced composites was investigated, and it was reported that a NaOH level of 5% provided improved bonding between surfaces and decreased fiber deterioration, resulting in superior mechanical characteristics [41]. The impact of immersion duration on *Gigantochloa scortechinii* fiber was investigated using the response surface approach. The findings revealed that the maximum yield strength was reached at 4 wt.% alkali-solution and a 12 h immersion duration [45]. Chemical treatment of the fiber improves the surface characteristics by breaking hydrogen bonding in their molecular structure [46]. The NaOH treatment resulted in the improved thermal stability of both hemp/palmyra multi-layered composites [47] and wood/hemp fiber-reinforced polypropylene composites [48]. The storage modulus of NaOH-treated sisal fiber-reinforced PLA composite increased significantly, exceeding the storage moduli of sisal fiber vinyl ester-based composites by 197.51% and sisal fiber-reinforced polyester composites by 288%. This significant increase in storage modulus is due to the reinforcement–resin interaction at the interface, which effectively constrains the atomic lattice movement of the molecular chains inside the composite system [34].

One investigation focused on exploring the dielectric properties of composites reinforced with rice husk fibers. It revealed fluctuations in the dielectric constant within the range of 6.32 to 8.742%. The dissipation factor of a composite containing 36% rice husk fibers increased to 0.068 at lower frequencies. Furthermore, an enhancement of 0.084% was reported in the lower frequency range for modified rice husk-reinforced polymer composites [24]. This phenomenon suggests that at the composite interface, the motion of polymeric chains decreases the dielectric losses in the rice husk-reinforced composite due to the effective interlocking between the matrix and fibers [30,49]. The research revealed the influence of NaOH on kenaf fiber’s sound absorption properties across various concentrations. The research found that the highest noise reduction coefficient was achieved by specimens modified with NaOH [50]. Moreover, a reduction in diameter corresponded to an increase in the fiber quantity per unit area, maintaining similar volume and thickness across samples. As a result, this enhancement increased each specimen’s surface contact area, which raised the coefficients of sound absorption throughout the frequency range. This analysis recommends employing a 6% NaOH concentration attains maximum sound absorption performance [36,51].

Another investigation focused on the hydrothermal characteristics of *Pterocarpus angolensis* fiber-reinforced PLA composites. The activation energy was optimized, reaching its highest value at 104 kJmol^−1^ for the laccase-reinforced PLA composites [52]. The water absorption rate varied among the different composites, with PLA demonstrating the least absorption at 0.54%. Following PLA, the absorption rates increased progressively for the alkaline-laccase, laccase, alkaline, and unprocessed composites. Additionally, thickness swelling was evaluated, revealing that PLA exhibited the minimum expansion at 1.54%, trailed by the alkali-laccase treatment of composite materials at 1.98% [23,53]. One study explored the influence of alkaline treatment on the biodegradation characteristics of *Cymbopogan citratus* fiber-reinforced cassava starch–palm composite [54]. It was evident that an extended burial duration led to increased weight loss across all composites, signifying a higher microbial presence within the materials. Moreover, this observation was potentially linked to the physical attributes of the raw fibers, which exhibited higher moisture absorption compared to the surface-modified fibers. Consequently, this increased water absorption rendered the composite more susceptible to micro-organism assaults, particularly targeting the raw specimens when exposed to water [55,56].

The application of TMMS significantly increased the thermal and hygro-thermal properties of the fiber-reinforced polymer composites [57]. Meanwhile, NaOH treatment may result in a reduction of certain crucial elements within *Hibiscus tiliaceus* fibers. Silane treatment has the potential to augment the SiO_2_ content within the fibers, which might enhance the bonding between the fibers and the resin [58]. Corn-stalk fibers were soaked in chemical mixtures containing varying loading levels of silane solution. Subsequently, the treated corn-stalk fibers underwent a moisture-removal test at 90 °C for 24 h. The findings indicated that corn-stalk fibers treated with 5 wt.% silane exhibited the maximum tensile strength of 223.33 ± 41.22 MPa, along with a Young’s modulus of 18.96 ± 2.43 GPa [59]. Additionally, the silane coupling agents improved the outer-layer interaction between the reinforcement and the resin [60]. The flexural properties of flax fibers were enhanced via a two-step modification process involving 2,2,6,6-tetramethylpiperidine-1-oxy radical-mediated oxidation, followed by amino-silane grafting. Interestingly, the composite treated with oxidation followed by silane treatment demonstrated an improvement in flexural properties. In contrast, the composite treated with pre-hydrolyzed silane exhibited a decrease in properties [61]. This discrepancy could potentially be attributed to the degradation mechanism, affecting the effective transfer of stress between the reinforcement and the resin within the composite material [62].

After silane treatment, banana fiber composites improved their α cellulose from 63.40% to 82.23%. The surface of bamboo fibers that underwent treatment using silane coupling agents then had amino, epoxy, and methyl functional groups applied to improve the surface roughness of the fibers. Notably, employing a 5 wt.% methyl functional group treatment resulted in enhanced tensile and flexural strengths, measuring 36.12 and 54.73 MPa, respectively. These values were 15.43% and 23.67% higher than those observed in raw bamboo fiber-reinforced polypropylene composites. Moreover, the thermal stability was raised from 467.93 °C to 470.62 °C upon employing the methyl functional group treatment on bamboo fibers. Additionally, this treatment induced an increase in crystallization temperature by 1.7 °C, while leading to a reduction in crystallinity by 5.8% in the resulting composite material [63].

The study investigated the effect of different silane coupling agents specifically, 3-amino-propyl-trimethoxy silane, γ-glycidoxy-propyl-trimethoxy silane, γ-metha-cryloxy-propyl-trimethoxy silane, and γ-mercapto-propyl-trimethoxy silane on the interface compatibility and characteristics of wheat straw-reinforced PLA composites. Among these, the γ-metha-cryloxy-propyl-trimethoxy silane-modified composite exhibited superior mechanical characteristics and enhanced moisture intake. Strain analysis revealed that within the linear visco-elastic range, the storage modulus of the treated composites surpassed that of the raw ones [64]. Additionally, the frequency range indicated that the storage modulus and complex viscosity of the treated composites were notably higher compared to the unmodified ones [35]. Both strain and frequency analyses emphasized the effectiveness of silane modification in improving the interfacial compatibility of the composite materials [65,66]. This study focuses on examining the dielectric properties of phenolic hybrid composites reinforced with silane-treated pineapple leaf and kenaf fibers, utilizing dielectric spectroscopy over a frequency range of 0.1 Hz to 1 MHz and temperatures spanning from 50 to 180 °C. This analysis revealed two distinct interfacial polarizations associated with the Maxwell–Wagner–Sillars effect. Remarkably, the silane coupling agents demonstrate an improved surface interaction between the fibers and resin, indicating the potential for developing high-performance composites suitable for industrial applications [35]. This outcome can be attributed to the elimination of a significant portion of hemicelluloses, lignin, and hydroxyl groups from the fibers’ outer layer. This treatment effectively diminished the water absorption behavior of fibers, thereby augmenting the potential for establishing improved surface interaction between the reinforcement and the resin [66].

The existing literature demonstrated that common chemicals can significantly impact the mechanical and thermal characteristics of PLA composites. However, the application of HRFs in PLA composites, when subjected to chemical treatments and a wide range of characterization techniques, has not been extensively explored. This experimental work attempted to fill this research gap by investigating the influence of chemically treated HRFs on the results of PLA composites. A novel aspect of this research is the application of both NaOH and TMMS treatments to optimize fiber–matrix adhesion and enhance composite properties. The innovation lies in the comprehensive evaluation of mechanical, thermal, dielectric, and acoustic behavior, along with the use of a less-explored plant fibers, to develop more sustainable and eco-friendly composites. This research also aims to develop high-performance composites that are suitable for futuristic applications, fostering a greener and more sustainable future.

## 2. Materials and Methods

### 2.1. Materials

The stems of *Hibiscus rosa-sinensis* plants were collected from Salem District of Tamil Nadu, India, and the fibers extracted from stems served as the primary plant material in this study. The chemical agents utilized in this research, including NaOH, polyvinyl alcohol (PVA), and TMMS, were purchased from Sigma-Aldrich, Karnataka, India. TMMS was utilized as a coupling agent to enhance the compatibility of fiber with the matrix. In addition, the PLA, a biopolymer used in this work, was bought from NatureWorks under the brand name Ingeo (NatureWorks, Chennai, India), specifically the biopolymer 2003D grade, known for its suitability in various biopolymer applications.

### 2.2. Extraction of HRFs

**Figure 1:**
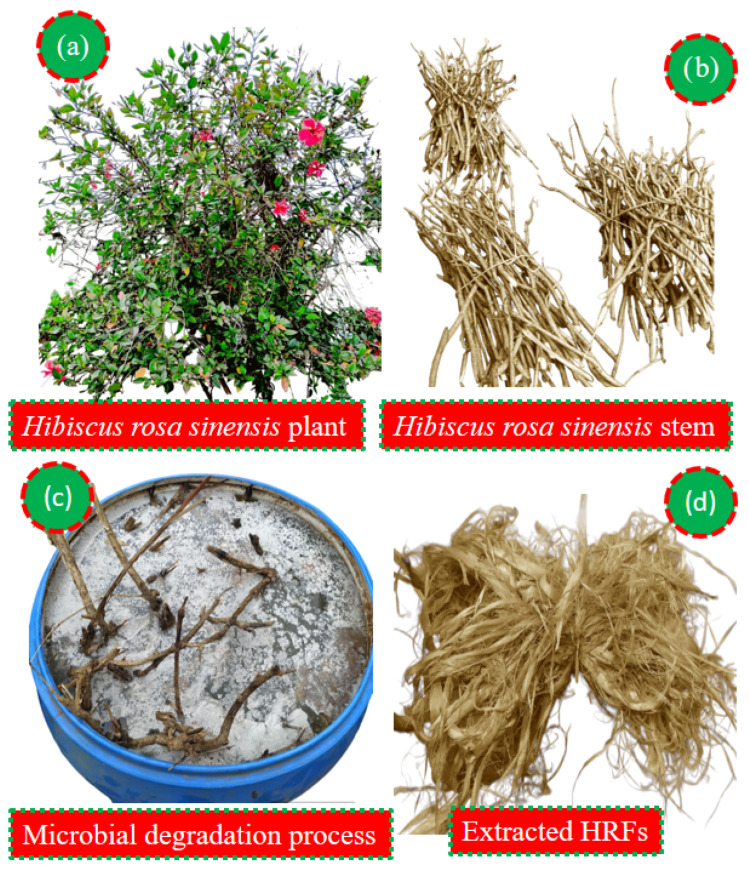
Extraction of HRFs.

The fibers from *Hibiscus rosa-sinensis* plants were extracted using the microbial degradation process by submerging the stems in water for 13 days (Figure 1). This method applies natural bacterial and fungal action to break down the pectin and lignin that bind the fibers to the woody core. During the retting period, enzymatic reactions occur, effectively loosening the fibers, allowing for their easy separation. The 13-day duration allows for optimal fiber quality and yield, ensuring that the fibers are adequately softened without becoming over-deteriorated [67]. Finally, the extracted fibers of 3 kg were dried under sunlight for 48 h and bundled for reinforcing into PLA.

### 2.3. Chemical Treatment of HRFs

The aim of chemical treatment is to enhance the properties and make the fibers more suitable for specific applications [29]. The process involves subjecting the HRFs to various chemical agents in order to remove impurities, break down lignin and pectin, and improve fiber flexibility and strength. This process can modify the fiber surface characteristics, making the fiber more receptive to dyes and other finishing treatments. Additionally, chemical treatment can increase the fiber resistance to microbial attacks and improve its overall durability. The goal is to optimize the fibers’ performance and expand their potential use in industries like textiles and paper manufacturing, while also contributing to the sustainable utilization of *Hibiscus rosa-sinensis* as a valuable natural resource [67,68].

#### 2.3.1. NaOH Treatment

The NaOH treatment involves a complex mechanism known as alkaline hydrolysis. When the fibers are immersed in the 5% NaOH solution, the alkali agents penetrate the cell walls and initiate a series of chemical reactions [27,69,70]. Acetyl groups in hemicellulose are cleaved off the hemicellulose chains as a result of a reaction between the hydroxide ions from the NaOH solution and the acetyl groups [40]. As a result, the hemicellulose breaks down into smaller fibers, which are soluble in the alkaline solution. In this research, NaOH reacts with the ester linkages in the lignin, causing the lignin to undergo saponification. It involves the breakdown of ester linkages in lignin into water-soluble salts and glycerol. This process allows the lignin to separate from the cellulose fibers as it loses its binding ability [71,72].

**Figure 2:**
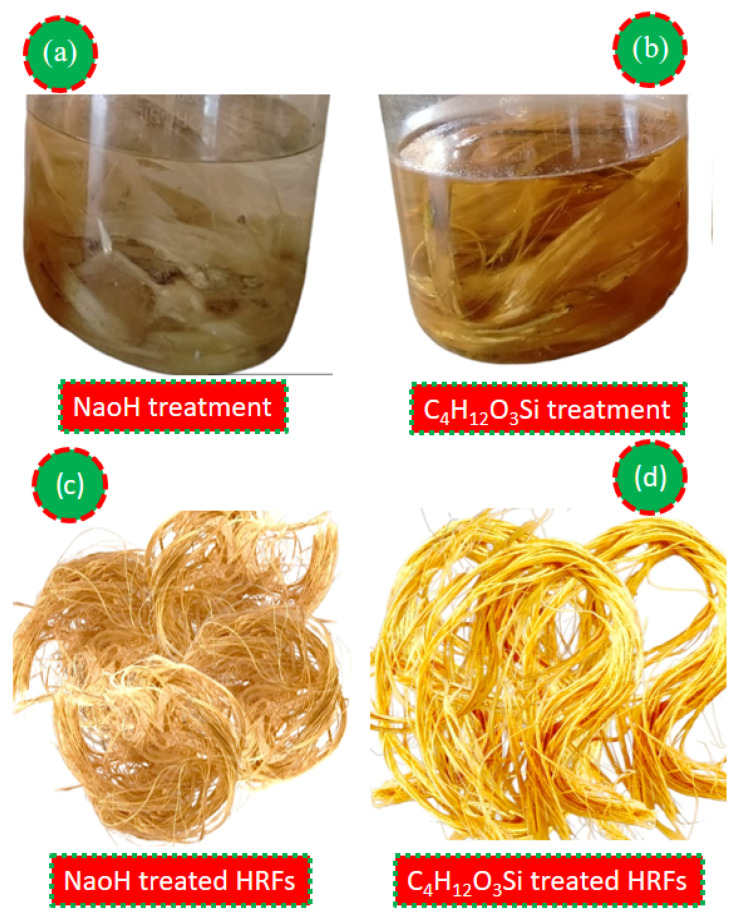
Surface-modification process of HRFs.

During the 72 h soaking period, the alkaline hydrolysis continues, and the NaOH solution gradually permeates into the fiber, further breaking down the lignin and pectin [73,74]. The hydrolysis of pectin, a polysaccharide that acts as a cementing agent in the fiber structure, results in the detachment of individual fibers. As the soaking time progresses, the NaOH treatment effectively removes impurities and non-cellulosic components, leaving behind purified cellulose fibers (Figure 2a). The resulting HRFs exhibit increased flexibility, enhanced tensile strength, and improved suitability for various industrial applications, making the NaOH treatment a critical step in optimizing the fiber properties for commercial use (Figure 2c).

#### 2.3.2. TMMS Treatment

The hydrophilic nature of HRFs poses a significant challenge in their extensive application, particularly in hydrophobic polymeric matrices, leading to the reduced adhesion and mechanical performance of the resulting composite materials [27]. As a solution, a vital chemical treatment process known as the chemical interfacial adhesion technique is employed to enhance surface interactions [74]. TMMS chemical compounds, which are hydrophilic elements with a Si atom attached to various functional bonds, have proven effective in this regard [75]. One end of the silane elements interacts with the resin, while the other end reacts with the hydrophilic fiber, effectively bridging the two materials [76]. The chemical reaction between 5% alkoxysilanes and the HRFs’ surface involves hydrolysis. During the subsequent drying or curing stage, a covalent linkage is formed with the substrate, leading to the creation of a strong and durable bond. This treatment enhanced the surface interactions between HRFs and the resin, resulting in improved mechanical properties.

In one study, a TMMS coupling agent with a chemical formula of CH_3_Si(OCH_3_)_3_ and a molar mass of 136.22 g/mol was used. The density of the silane is 955 kg/m^3^, and its boiling point ranges between 102 and 104 °C. To evaluate the effects of silane treatment, HRFs underwent treatment with a solution comprising a 5 wt.% blend of the ethanol and water [32]. Before application, the silane was pre-hydrolyzed at ambient conditions for 2 h in an 70/30 vol. % mixture of ethanol and water to create active silanol groups [27]. Subsequently, the fibers were immersed in the presence of a catalyst solution for 2 h, utilizing a ratio of 22.5 mL/g between the vol. of the solution and the weight of the fiber. Then the HRFs were warmed in an oven at 72 °C for 18 h, ensuring the complete removal of moisture and the formation of strong covalent bonds between the TMMS and the hydroxyl groups on the reinforcement outer layer [38]. This TMMS process improves the interfacial adhesion between the hydrophilic HRF and hydrophobic matrices, ultimately improving the mechanical properties of the composite materials. This covalent linkage significantly enhances the interfacial adhesion between the hydrophilic fiber and the hydrophobic matrix, such as polymeric materials, in composite applications (Figure 2b).

Additionally, the silane treatment acts as a surface modifier, imparting hydrophobic characteristics to the hydrophilic fiber. The addition of TMMS to the HRFs reduces their affinity to water, thereby addressing the adhesion issues associated with hydrophilic fibers in hydrophobic matrices. The hydrolyzed silane forms an exterior coating on the HRFs’ outer layer, minimizing water uptake and promoting better sustainability with the hydrophobic resin (Figure 2d). This hydrophobic modification induces enhanced resistance to surrounding conditions and enhances the life cycle of the product and durability of the composite material. The mechanism of linking through TMMS treatment demonstrates its significance in optimizing the interfacial adhesion and performance of HRF samples, making it a valuable approach for various industrial applications in composite material development.

### 2.4. Fabrication of HRF Laminates

**Figure 3:**
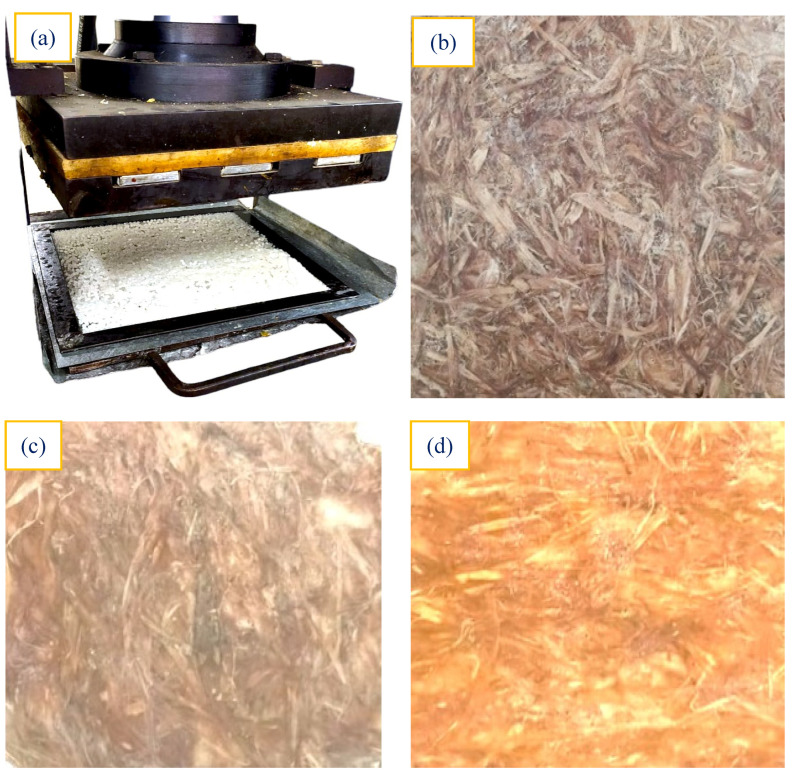
Fabrication of HRFCs. (**a**) Compression molding setup, (**b**) UHRFC laminate, (**c**) NHRFC laminate and (**d**) THRFC laminate.

In this research, three distinct configurations of HRF/PLA composites were fabricated and investigated. The laminates comprised untreated, NaOH-treated, and TMMS-treated HRFs as reinforcement with the PLA matrix in a ratio of 1:1. Before composite fabrication, the HRFs were dried to ensure their mechanical integrity and uniformity. To manufacture the composites, a compression molding machine and rectangular glass molds measuring 300 × 270 mm were used (Figure 3a). To prevent adhesion during the process, a PVA releasing gel was applied to the mold exterior areas. The PLA and curing agent were completely blended and degassed to remove any trapped air. The infusion of catalyzed resin into the fiber stacks was followed by curing at ambient conditions for 24 h. To maintain consistency, all composites were produced with a standardized 50% (±1) fiber weight fraction (Figure 3b–d). This controlled and systematic approach allowed for an evaluation of the mechanical characteristics and performance of the different configurations, shedding light on the effects of HRFs’ processing on the overall performance of HRF/PLA composites and their sustainable utility.

### 2.5. Mechanical Testing

#### 2.5.1. Tensile Test

**Figure 4:**
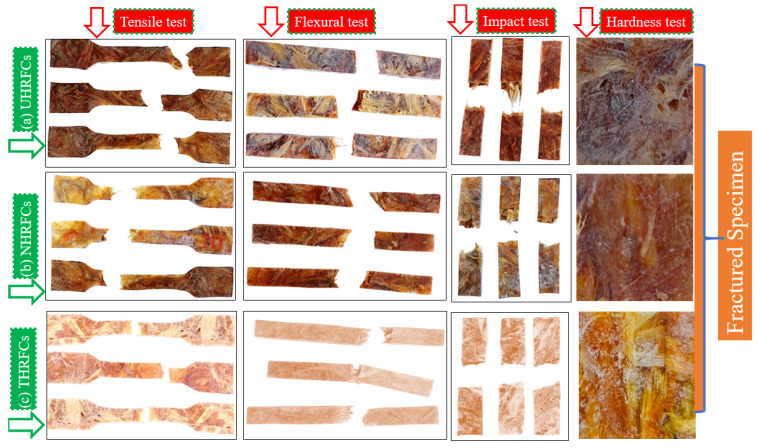
Fractured specimen subjected to mechanical loading.

The tensile test specimens were prepared as per the standard ASTM D638, with specific dimensions, including a gauge length of 60 mm, width of 19 ± 0.3 mm, and thickness of 3 ± 0.2 mm. The specimens are oriented to ensure that the loading direction aligns with the HRF orientation and to minimize any potential effects of holding. The tensile experiment is carried out on the universal testing machine (UTM; make, FIE; Model, UTN 40; S. No. 11/98-2451), applying a controlled cross-head speed of 2.1 mm/min, and the load and elongation are continuously recorded. The test repeated for three specimens and the mean results are discussed. This procedure allows for the accurate evaluation of tensile strength, modulus, and elongation. Fractured tensile-tested specimens are presented in Figure 4.

#### 2.5.2. Flexural Test

In accordance with ASTM D790, specimens of size 144 ± 0.2 mm long, 15 ± 0.05 mm width, and 3 ± 0.15 mm thick were prepared. The experimental setup involved positioning the specimen between supports, spaced at a span of 144 mm. To ensure controlled and consistent conditions, the jaws of the testing apparatus were programmed to operate at a speed of 0.73 mm/min. By effectively evaluating the material’s flexural characteristics, a three-point bending test offered more accurate results of how the material behaves mechanically under bending load. The fractured flexural test specimens are presented in Figure 4.

#### 2.5.3. Impact Test

Adhering to standard ASTM D256, the experimental setup was devised to ensure precision, consistency, and the reliability of results. Specimens with dimensions measuring 63.5 ± 0.3 mm in length, 12.7 ± 0.2 mm in width, and 3 ± 0.2 mm in thickness were prepared for the Izod impact test. The samples were loaded to a pendulum apparatus, and the striking tip was aligned with the mid-length of the specimen. The pendulum was released from a pre-defined height, inducing a sudden impact force on the specimen. The absorbed energy and subsequent fracture behavior were then observed. The fractured impact-test specimens are presented in Figure 4.

#### 2.5.4. Hardness Test

Hardness test specimens were prepared based on the ASTM D2240 standard, with a thickness of 6 mm to accommodate the Shore-D hardness tester. A durometer was used to exert a defined indentation onto the specimen surface, and several readings were obtained at various points on each sample. This procedure enabled the quantification of the material’s hardness, shedding light on its resistance to localized deformation and providing essential insights into its overall mechanical behavior. The tested hardness specimens are given in Figure 4.

### 2.6. Thermo-Gravimetry Study

Confirming to the ASTM E1131 standard, a Q500 instrument from TA instruments (New Castle, DE, USA) was used to do the experiments. Specimens of known mass were subjected to a heating rate of 10 °C/min. A platinum pan containing about 25 mg of each specimen was treated under nitrogen, from ambient heating to 600 °C, while the mass loss of the samples was directly proportional to the heating rate. The analysis permitted the detection of moisture evaporation, deterioration, and the start of breakdown. The generated TGA curves offered information on the material’s thermal stability, decomposition rates, and possible degradation temperatures. By describing the complexities of the TGA procedure, this study increases our understanding of the thermal behavior of HRFCs, contributing to their effective utilization across industries.

### 2.7. Visco-Elastic Properties

The assessment of visco-elastic properties through dynamic mechanical analysis (DMA) provided us insight into the time-dependent mechanical properties of HRFCs. Adhering to the ASTM D4065 standard, the experiment was designed to ensure precision, consistency, and reliable outcomes. Specimens of dimensions 35 × 13 mm were prepared to suit the TA Q800 DMA apparatus, a controlled sinusoidal strain input of 0.1% was applied over a range of frequencies fixed at 1 Hz, and the heating rate was 2 °C/min for all temperatures ranges.

### 2.8. Dielectric Properties

Dielectric spectroscopy provides detailed information about composites’ electrical characteristics and polarization mechanisms. The circular test specimens, measuring 13 mm in diameter, were examined using the LCR Hi-tester 3500, made by HIOKI in Nagano, Japan. Following ASTM D150 guidelines, the tests included exposing the samples to frequencies between 50 Hz and 50 MHz while maintaining a 10 mA current. Frequency sweeps were carried out over a pre-determined range, and complicated permittivity and impedance data were obtained. These measurements enabled the evaluation of the material’s capacitance, conductivity, and polarization behavior, providing critical information on its electrical response under different conditions.

### 2.9. Sound Absorption Study

This study was used to investigate the sound absorption characteristics, acoustic behavior, and energy dissipation mechanisms of composite materials. The acoustic properties of HFRCs were investigated using the impedance tube method, specifically adopting the two-microphone transfer-function approach described in ISO 10534-2, developed for horizontally placed samples. In this study, a medium-pressure Bruel and Kjaer Model 4206A impedance tube was used to evaluate acoustic characteristics from the range of 125 to 5000 Hz. A container with a 63.5 mm tube, suited for the circular samples with identical diameters, was used. The noise reduction coefficients of the HRFCs were assessed following ASTM 1050 standards, using the impedance tube method. These data allowed for an assessment of the specimen’s ability to attenuate sound at various frequencies, providing information on its acoustic performance.

### 2.10. Water Absorption Test

Our investigation on water absorption properties elucidated the moisture uptake and swelling behavior of composites. The water absorption test was carried out using distilled water in accordance with the ASTM D570 standards. Initially, specimens sized 200 × 25 × 3.5 mm underwent oven drying at 80 °C until reaching a consistent weight. These dried specimens were immersed in pH-neutralized water at ambient conditions. The measurement of water absorption and thickness swelling occurred at 24 h intervals until saturation was reached. After each interval, the samples were taken out from the water, dried by wiping, and measured using an analytical instrument with a precision of 0.01 grams. To ensure accuracy, five specimens of each composite type underwent testing, and the average measurements were recorded.

### 2.11. Biodegradability Study

The evaluation of biodegradability properties through a controlled degradation atmosphere helped shed light on the environmental damage and potential eco-friendly disposal of composites. Specimens were prepared and subjected to simulated degradation conditions, typically in composting environments. Adhering to the standard ASTM D6400, the experiment was conducted to ensure precision, consistency, and reliable outcomes. The degradation process was monitored by measuring weight loss. These measurements allowed for the quantification of the specimen’s biodegradation rate and extent, revealing its ability to break down and contribute to reduced environmental impact.

### 2.12. Morphological Study

To conduct SEM analysis, the samples were made into small pieces, and then a gold-sputtering process was applied to coat the surface of the samples before examination using a microscope. The objective was to investigate the adhesion between the reinforcement and resin on the chemically treated outer layer, utilizing a microscope of model SIGMA and make Carl Zeiss Microscopy Ltd. (New York, NY, USA). The microscope operated within a magnification range from ×500 to a maximum operational voltage of 10 kV. The energy level of the electron beam used to scan the sample in the microscope with electron high tension of 5 kV provided better resolution and deeper penetration into the sample.

## 3. Results and Discussion

### 3.1. Mechanical Strength Analysis

#### 3.1.1. Tensile Strength

**Figure 5:**
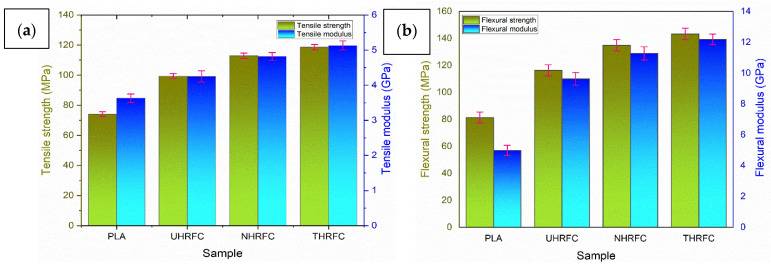
(**a**) Tensile strength and modulus. (**b**) Flexural strength and modulus of HRFCs.

The tensile behavior of the HRFCs was evaluated, and the results are shown in Figure 5a. Initially, PLA had a tensile strength of 74.08 ± 0.66 MPa. From the results, it is found that the UHRFCs showed a significant increase in tensile strength (99.36 ± 0.73 MPa), indicating a reinforcement effect. The NaOH treatment improved the fiber–matrix interaction and interfacial adhesion in NHRFCs, resulting in a tensile strength increase of 112.93 ± 0.77 MPa. THRFCs showed a peak tensile strength of 118.62 ± 0.83 MPa after TMMS treatment, due to improved fiber–matrix interaction. The 13.66% increase in NHRFCs over UHRFCs indicates a significant effect of NaOH treatment. Similarly, the 19.39% rise in THRFCs over UHRFCs demonstrates the higher efficacy of TMMS treatment. The 5.03% rise in THRFCs over NHRFCs indicates that, while TMMS treatment improves tensile strength, the effect is significantly lesser than the leap from UHRFCs to THRFCs. These findings confirm the important influence of both treatments in boosting tensile strength, with THRFCs showing a somewhat higher tensile strength than NHRFCs.

#### 3.1.2. Tensile Modulus

The analysis outlines the mechanical behavior of the tested HRFCs a perceptible increasing trend is found throughout the various sample compositions. The raw PLA composite has an elemental tensile modulus of 3.63 ± 0.72 GPa, which provides insight into the dataset’s inherent dispersion. UHRFCs’ tensile modulus significantly improved to 4.25 ± 0.76 GPa. The NHRFCs resulted in having an increased tensile modulus of 4.82 ± 0.81 GPa. THRFCs had the highest tensile modulus of 5.13 ± 0.83 GPa, marking the effect of the surface treatment. The observed increase in tensile modulus among composites indicates that surface treatments affect material stiffness. The 13.41% increase in tensile modulus for NHRFCs over UHRFCs demonstrates the way NaOH treatment increases the stiffness of the composites. This can be due to better adhesion at the fiber–matrix interface, which is caused by surface modifications made possible by NaOH treatment. Similarly, the 20.70% improvement in tensile modulus for THRFCs over UHRFCs shows the effect of TMMS treatment. The silane treatment improves interfacial bonding and fiber–matrix adhesion, which leads to higher stiffness. The 6.43% increase in THRFCs over NHRFCs reveals that, while the TMMS treatment continues to enhance stiffness, its influence on tensile modulus is slightly lower than that from UHRFCs to THRFCs. These findings indicate the importance of both treatments in increasing tensile modulus, with THRFCs outperforming NHRFCs in terms of stiffness. This study’s significant addition is that the synergistic effects of TMMS treatment produce more than NaOH alone, thereby bringing new insights into optimizing fiber treatments for greater impact performance. These results are comparable to the results obtained by other researchers [59].

#### 3.1.3. Flexural Strength

The determination of flexural strength values and their accompanying standard deviations verified that the results are reliable and consistent. UHRFCs outperformed PLA in respect to flexural strength, yielding 116.36 ± 4.11 MPa on average (Figure 5b). NHRFCs demonstrated improved flexural characteristics, with a mean strength of 134.96 ± 4.21 MPa. THRFCs demonstrated the highest flexural strength, with a mean value of 143.33 ± 4.22 MPa. The 15.98% improvement in flexural strength of NHRFCs over UHRFCs demonstrates the effect of NaOH treatment on the material’s capacity to carry bending loads. This improvement indicates increased interfacial bonding between the reinforcement and the resin as a result of the NaOH treatment, allowing for better load distribution across the material. Furthermore, the 23.17% increase in flexural strength of THRFCs over UHRFCs indicates a stronger effect of the TMMS treatment [59]. Silane treatment improves interfacial bonding and fiber–matrix adhesion, hence increasing the composite’s capacity to withstand bending loads. The 6.20% rise in THRFCs over NHRFCs indicates that, while the TMMS treatment improves flexural strength, its effect is significantly lesser than the increase from UHRFCs to THRFCs. These data confirm that both treatments significantly contribute for the improvement of flexural strength, with THRFCs showing a slightly more pronounced improvement than NHRFCs.

#### 3.1.4. Flexural Modulus

This study analyzes the flexural modulus of a pure PLA and HRFCs treated with different chemical agents. The UHRFCs had a significantly higher flexural modulus than pure PLA, with an average of 9.63 ± 0.41 GPa. The NHRFCs demonstrated further improvements, with a mean flexural modulus of 11.28 ± 0.43 GPa. THRFCs had the highest flexural modulus, measuring 12.19 ± 0.34 GPa on average. The 17.13% increase in flexural modulus of NHRFCs over UHRFCs indicates that NaOH treatment improves the composite resistance to deformation under bending stresses. This enhancement indicates that the treatment improves adhesion between the HRF and the PLA resin, resulting in a stronger material with increased bending resistance. Furthermore, THRFCs have a 26.58% higher flexural modulus than UHRFCs, indicating that the TMMS treatment has a greater influence on composite stiffness. This treatment considerably improves interfacial bonding and fiber–matrix adhesion, resulting in a much stronger composite material. The 8.06% rise in THRFCs over NHRFCs indicates that, while the TMMS treatment continues to improve flexural modulus, its effect is significantly less when compared to the increase from UHRFCs to THRFCs. This result highlights the considerable contributions of both treatments in strengthening flexural modulus, with THRFCs showing slightly more pronounced improvement than NHRFCs.

#### 3.1.5. Impact Strength

**Figure 6:**
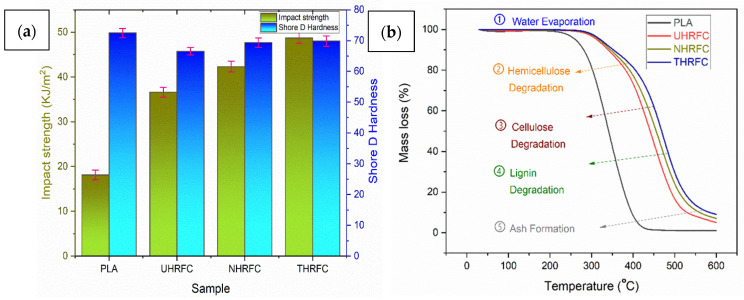
(**a**) Impact strength and Shore-D hardness; and (**b**) mass loss analysis of HFRCs.

Figure 6a shows the impact strength values for HRFCs. From the figure, it is observed that UHRFCs had a much higher impact strength than pure PLA, with an average of 36.61 ± 1.11 KJ/m^2^. The NHRFCs showed improved impact resistance, whereas the THRFCs had the maximum impact strength of 48.72 ± 1.22 KJ/m^2^. The 15.62% increase in impact strength of NHRFCs over UHRFCs indicates that NaOH treatment improves the composite’s ability to withstand sudden impacts without fracturing. This improvement indicates an increase in the composite’s toughness due to increased adhesion between the fiber and the PLA matrix. The treatment potentially modifies the surface chemistry of the HRFs, facilitating more efficient stress transfer and energy dissipation upon impact. Furthermore, the 33.07% increase in impact strength of THRFCs compared to UHRFCs demonstrates the significant effect of the TMMS treatment in improving the composite’s impact resistance. Furthermore, the 15.09% increase in THRFCs over NHRFCs shows that, while both treatments significantly improve impact strength, TMMS has a slightly stronger effect on this property than the NaOH treatment. It demonstrates that the TMMS treatment significantly improves the composite’s ability to withstand sudden shocks and absorb more energy before failure.

#### 3.1.6. Shore-D Hardness

Shore-D hardness and the associated standard deviations were measured in order to ensure the precision and reliability of the results. The UHRFC showed a significant hardness level, but slightly lower than that of pure PLA, with a mean hardness value of 66.54 ± 1.23 (Figure 6a). Furthermore, the NHRFCs had better hardness characteristics, while the THRFCs revealed comparable Shore-D hardness, highlighting the various influences of chemical treatments on material hardness behavior. The 4.19% increase in Shore-D hardness of NHRFCs over UHRFCs demonstrates the significant role of NaOH treatment in improving the composite’s surface hardness. Similarly, the 5% increase in Shore-D hardness of THRFCs over UHRFCs demonstrates the significant influence of the TMMS treatment on improving the HRFCs’ hardness. Furthermore, the 0.77% improvement in THRFCs over NHRFCs indicates that, while both treatments significantly improve Shore-D hardness, the TMMS treatment has a slightly stronger effect than the NaOH treatment. This indicates that the TMMS treatment significantly contributes to the composite’s surface hardness and stiffness.

### 3.2. Thermo-Gravimetry Analysis

A thorough evaluation of the differential thermo-gravimetry (DTG) characteristics reveals the specific thermal degradation behavior of the materials under testing. The initial decomposition temperatures for PLA (97.45 ± 3.62 °C), UHRFCs (98.66 ± 4.13 °C), NHRFCs (98.13 ± 3.22 °C), and THRFCs (99.99 ± 2.11 °C) show a consistent improvement in the thermal stability of HRFCs compared to pure PLA (Table 1). The onset temperatures of UHRFCs (304.15 ± 4.23 °C), NHRFCs (309.89 ± 3.63 °C), and THRFCs (312.99 ± 3.96 °C) are higher than for PLA (292.61 ± 4.15 °C). The results showed that the THRFCs (469.07 ± 3.11 °C) have the highest thermal stability. Composites with higher residue percentages (UHRFCs, 7.44 ± 0.06%; NHRFCs, 8.56 ± 0.03%; THRFCs, 9.71 ± 0.01%) compared to PLA (5.25 ± 0.09%) show superior resistance to thermal decomposition, making them suitable for high-temperature applications. THRFCs have the highest activation energy (186.75 ± 1.99 kJ/mol), making them ideal for demanding applications requiring exceptional thermal stability.

The examination of the mass loss (%) results presented in Figure 6b shows that UHRFCs, NHRFCs, and THRFCs have significantly improved thermal stability when compared to pure PLA. The reduction in mass loss indicates their great resistance to thermal degradation, an important feature for applications at high temperatures. Notably, the higher residue percentages reported in NHRFCs and THRFCs highlight their extraordinary capacity to generate char, implying prospective applications in fire-resistant and high-temperature manufacturing environments. The activation energy levels, which are particularly high in THRFCs, support HRFCs’ superior thermal stability. The TGA study’s major conclusion is that the TMMS treatment considerably improves high-temperature performance, giving new ways for manufacturing thermally resistant eco-friendly materials.

**Figure 7:**
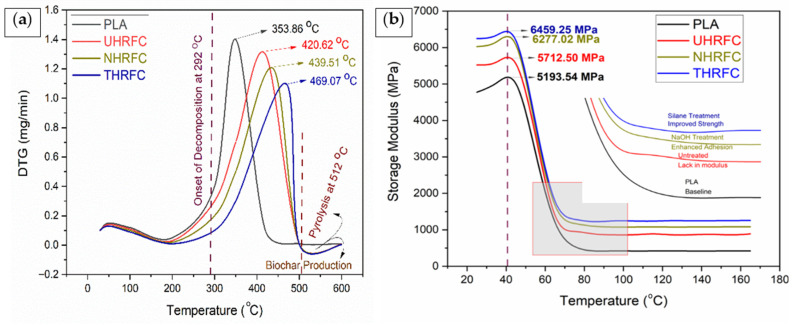
(**a**) DTG analysis and (**b**) storage modulus of HRFCs.

The DTG analysis of HRFCs is shown in Figure 7a. The figure shows that the NaOH treatment results in a 4.49% improvement in peak temperature and a 1.68% increase in activation energy in NHRFCs when compared to UHRFCs. These improvements indicate an increase in the composite’s thermal stability, implying a greater need for higher temperatures and more energy for thermal breakdown. The NaOH treatment improves interfacial bonding and cleans the fiber surface, which greatly contributes to the material’s thermal stability. Similarly, the 11.51% increase in peak temperature and 3.02% increase in activation energy noticed in THRFCs compared to UHRFCs indicate that TMMS treatment has an impactful influence on the material’s thermal properties. The treatment improves interfacial adhesion by modifying the HRF surface, which increases the HRFCs’ thermal stability. Furthermore, the 6.72% and 1.32% increases in peak temperature and activation energy found in THRFCs compared to NHRFCs demonstrate that TMMS treatment has a more significant effect than NaOH treatment. This shows that, while both treatments improve thermal stability, the effect of TMMS treatment on enhancing HRFC resistance to thermal degradation is significantly greater.

### 3.3. Visco-Elastic Behavior

#### 3.3.1. Storage Modulus

The storage modulus (E’) of PLA falls substantially as temperature increases (Figure 7b). This trend indicates that PLA becomes less stiff and more compliant as temperature rises. The decrease in E’ is normal for thermo-plastic materials, as higher temperatures allow for greater molecular mobility, lowering the material’s stiffness. UHRFCs follow the same tendency as PLA, with a reduction in E’ as temperature rises. The reduction in stiffness can be attributable to both the resin and the HRFs. NHRFCs also show a drop in E’ as temperature rises because the NaOH treatment may affect the composite’s visco-elastic characteristics. The considerable decline in E’ at increasing temperatures indicates that HRFCs become less stiff and more elastic. THRFCs exhibit a decrease in E’ as temperatures increase, similar to the behavior of other materials. The presence of TMMS on the HRF surface may affect the composite’s visco-elastic properties, but it still exhibits typical thermo-plastic behavior. NaOH treatment affects the HRF surface by eliminating impurities like lignin and hemicellulose and improving the HRF’s cleanliness. This procedure improves the interfacial adhesion between the HRF and the PLA, resulting in a more rigid composite structure. The observed 9.88% increase in E’ for NHRFCs over UHRFCs indicates increased stiffness due to enhanced fiber–matrix interaction. The NaOH treatment changes the HRF surface, resulting in more efficient load transfer between the fiber and the matrix, reinforcing the composite structure and increasing the E’. Similarly, the 13.07% increase is reported in THRFCs compared to UHRFCs demonstrates TMMS treatment’s significant influence on improving material stiffness. Furthermore, the 2.90% rise observed in THRFCs compared to NHRFCs indicate that the additional effect of TMMS treatment on the E’ is more substantial than NaOH treatment. It also indicates that, while both treatments increase material stiffness, the TMMS treatment has a significantly greater effect on enhancing composite rigidity. Overall, the increases in E’ for both treated composites show that the NaOH and TMMS treatments improve the materials’ stiffness and structural integrity.

#### 3.3.2. Loss Modulus

**Figure 8:**
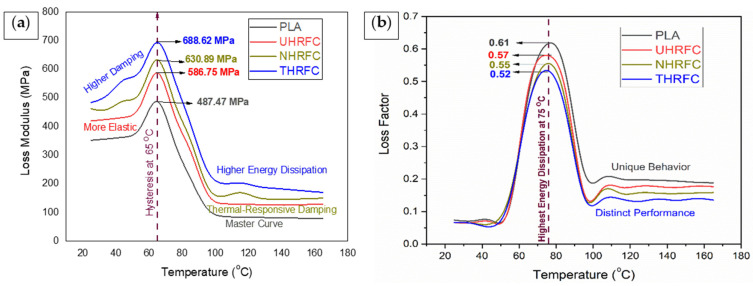
(**a**) Loss modulus and (**b**) loss factor analysis of HRFCs.

Figure 8a shows that PLA’s loss modulus (E”) gradually increases as temperature rises. This pattern is typical of thermos-plastic materials, suggesting increased visco-elasticity and energy dissipation as temperature rises. UHRFCs exhibit a consistent and significant increase in E” with increasing temperature. This implies that the composite becomes more visco-elastic at higher temperatures, which is advantageous for applications that require energy absorption and damping. NHRFCs have strong temperature sensitivity in their E” behavior. It rapidly increases as the temperature rises, indicating improved visco-elastic characteristics. This behavior is useful in applications where temperature-dependent dampening is required. THRFCs show a steady but significant increase in E” with increasing temperature.

The 7.52% and 17.36% increases in the E” of NHRFC and THRFC over UHRFCs, respectively, demonstrate that both treatments are beneficial in enhancing damping capabilities. The NaOH treatment largely removes contaminants and improves the cleanliness of the fiber surface, resulting in better energy dissipation inside the composite. The TMMS treatment, on the other hand, modifies the fiber surface even more, considerably increasing interfacial adhesion and, as a result, the composite’s energy dissipation capabilities. This results in a significantly greater E” compared to UHRFCs. Furthermore, the 9.15% increase in the E” of THRFCs over NHRFCs demonstrates the greater influence of TMMS treatment on improving the material’s damping performance. This shows that, while both treatments improve energy dissipation and damping, the TMMS treatment has a greater impact on the E”. Both NaOH and TMMS treatments effectively increase the loss modulus, indicating increased energy dissipation and damping properties in the treated composites. These treatments modify the fiber–matrix interface, promoting greater interfacial adhesion, which improves the materials’ ability to absorb energy and minimize vibrations.

#### 3.3.3. Loss Factor

Figure 8b shows that thermo-plastic materials exhibit an increasing loss factor (tan (δ)) with rising temperature, which is a common feature. It denotes the materials’ potential to display improved visco-elasticity and energy dissipation as they become more sensitive at higher temperatures. This characteristic is especially useful in applications that require dynamic loads, vibrations, and oscillations because it represents the materials’ capacity to efficiently absorb and disperse mechanical energy. The differences in tan (δ) among the studied materials are caused by a complex interaction of elements, such as chemical treatments and inherent material characteristics. Compared to pure PLA, all HRFCs exhibit improved visco-elastic behavior, with UHRFC consistently having the highest tan (δ) across temperatures. This shows that integrating HRFs and applying different treatments resulted in increased energy dissipation and visco-elasticity.

### 3.4. Dielectric Properties Analysis

#### 3.4.1. Dielectric Constant

**Figure 9:**
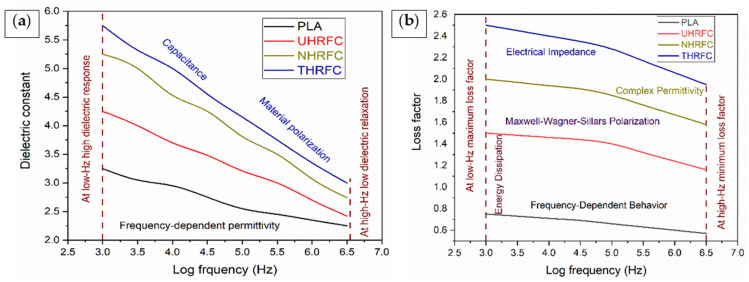
(**a**) Dielectric constant and (**b**) dielectric loss factor analysis of HRFCs.

The dielectric constant for pure PLA was 3.25 at the lowest frequency observed (log frequency = 3 Hz). UHRFCs had a higher dielectric constant of 4.25, followed by NHRFCs at 5.25 and THRFCs at 5.75, as is shown in Figure 9a. This indicates that the incorporation of HRFs, with the various treatments, results in a significant increase in the dielectric constant of HRFCs, as compared to pure PLA. The improved dielectric constants of HRFCs indicate high polarization and energy storage properties. At log frequency (6.5 Hz), pure PLA showed a dielectric constant of 2.25, with UHRFC at 2.42, NHRFC at 2.74, and THRFC at 3. NaOH treatment helps to remove impurities and superficial pollutants from the fiber surface, resulting in a cleaner, more polar surface. This treatment improves the contact between the fibers and matrix, enhancing the dielectric constant. The higher dielectric constant in the NaOH-treated composite, reflected by a 23.52% increase over untreated, shows an increased ability to store electrical energy when subjected to an electric field, most likely due to enhanced polarization at the fiber–matrix interface. Similarly, TMMS treatment increases the dielectric constant further. The significant increase in the dielectric constant, 35.29% above UHRFC, indicates an even larger potential to store electrical energy than the NaOH-treated composite. This implies that while both treatments augment the dielectric constant, the impact of TMMS treatment is more evident, most likely due to modification of the fiber surface and consequent improvement in interfacial bonding with the matrix.

NaOH treatment tends to improve the dielectric constant by increasing material polarization and capacitance. The treatment most likely removes contaminants from HRFs, resulting in a more polarized surface suitable for increased material polarization. This causes a significant increase in capacitance, particularly at lower frequencies, resulting in a stronger dielectric response at those frequencies. At low frequencies, materials exhibit an increased dielectric response due to greater polarization, suggesting the capacity to store energy efficiently in an electric field. In contrast, TMMS treatment improves the dielectric constant by improving the material’s dielectric properties. The functional groups produced during TMMS treatment promote stronger interfacial bonding and greater material polarization. At higher frequencies, this treatment reduces dielectric relaxation, indicating a more regulated response to electrical fields. NaOH and TMMS treatments affect the dielectric constant through different ways.

#### 3.4.2. Dielectric Loss Factor

At the lowest observed frequency (log frequency = 3 Hz), pure PLA had a loss factor of 0.75, as illustrated in Figure 9b. As we go on to HRFCs, a clear trend develops, indicating a steady increase in the loss factor. Figure 9b also shows that UHRFCs had a higher loss factor of 1.5, followed by NHRFC at 2, and then THRFC, with the highest loss factor of 2.5. This trend emphasizes the significant increase in the loss factor of HRFCs compared to pure PLA due to the surface treatments. The composites’ improved loss factor indicates their better ability to dissipate electrical energy as heat when subjected to alternating electric fields, an ability that is useful for applications involving electrical energy. As frequency increases, the loss factor of all materials decreases, which is consistent with typical dielectric material behavior. At a log frequency of 6.5 Hz, the loss factors were as follows: pure PLA = 0.57, UHRFCs = 1.16, NHRFCs = 1.58, and THRFCs = 1.95. This reduction in loss factor with higher frequencies represents improved electrical insulation characteristics at higher frequencies, which can be useful in some applications. This study advances the field by showing that TMMS treatment improves dielectric characteristics substantially more than NaOH treatment, providing new insights into enhancing the electrical performance of bio-based composites.

### 3.5. Sound Absorption Coefficient

**Figure 10:**
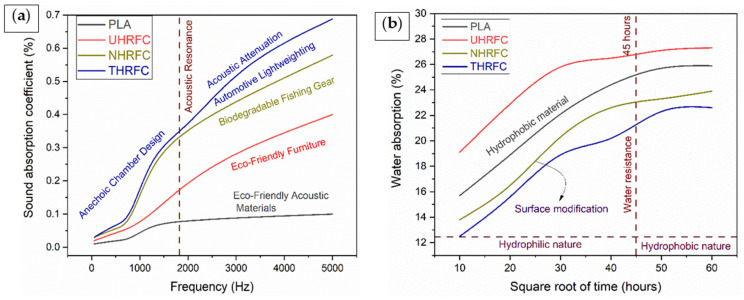
(**a**) Sound absorption coefficient and (**b**) water absorption analysis of HRFCs.

Figure 10a provides insights into the sound absorption characteristics at various frequencies. The sound absorption coefficient (%) clearly fluctuates with frequency, providing important information for understanding the acoustic absorption capabilities of various materials. At lower frequencies, PLA has the lowest sound absorption coefficient of 0.01%, indicating a limited ability to absorb sound waves. HRFCs, on the other hand, have significantly higher absorption coefficients, making them the best for absorbing sound in this frequency range. As the frequency increases between 500 and 800 Hz, all materials show a significant improvement in sound absorption. UHRFCs and NHRFCs surpass PLA, with THRFCs continuously having the highest sound absorption coefficients. This trend demonstrates that the incorporation of HRFs, together with the various treatments used, improves the composites’ acoustic absorption capacity. At higher frequencies, such as 1250 Hz, 2000 Hz, 3150 Hz, and 5000 Hz, the tendency persists, with THRFCs regularly outperforming other materials in sound absorption. NHRFCs also show considerable improvements, but PLA falls behind all composites. These findings highlight the importance of chemical treatments on natural fibers in improving their sound-absorbing properties and producing effective acoustic materials.

### 3.6. Water Absorption Analysis

The graph (Figure 10b) depicts the water absorption percentages of PLA and HRFCs at various time intervals. In the initial stage (at 10 h), UHRFCs have the highest water absorption percentage of 19.1%, followed by PLA (15.7%), NHRFCs (13.8%), and THRFCs. As the square root of time increases, all materials show an increased trend in water absorption %, indicating their ability to absorb water over time. UHRFC consistently retains the highest water absorption % during the investigated time intervals, a result that can be attributed to untreated HRFs’ natural hydrophilic character. On the other hand, PLA and THRFCs display reduced water absorption percentages, mostly owing to the inherently hydrophobic qualities of PLA and the probable water-repellent features of TMMS treatment in THRFCs.

### 3.7. Biodegradability Analysis

**Figure 11:**
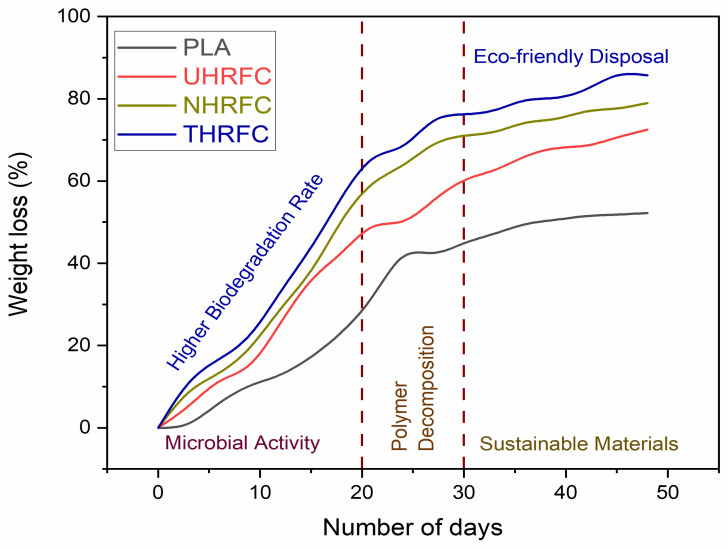
Biodegradability analysis of HRFCs.

In biodegradation analysis, the number of days is the independent variable on the x-axis, and the weight loss % is the dependent variable on the y-axis. The graph shown in Figure 11 depicts the link between time and the level of material degradation. The graph shows that, over the 48-day testing period, a gradual increase in weight loss for HRFCs occurs, emphasizing their biodegradability. In contrast, PLA loses little weight, showing that it is resistant to degradation. THRFC, defined by its rapid weight loss rate, emerges as a promising alternative for applications requiring accelerated biodegradation. This graphical representation clearly depicts the time progression of biodegradation in each substance, providing significant information about their environmental impact.

### 3.8. Morphological Analysis

**Figure 12:**
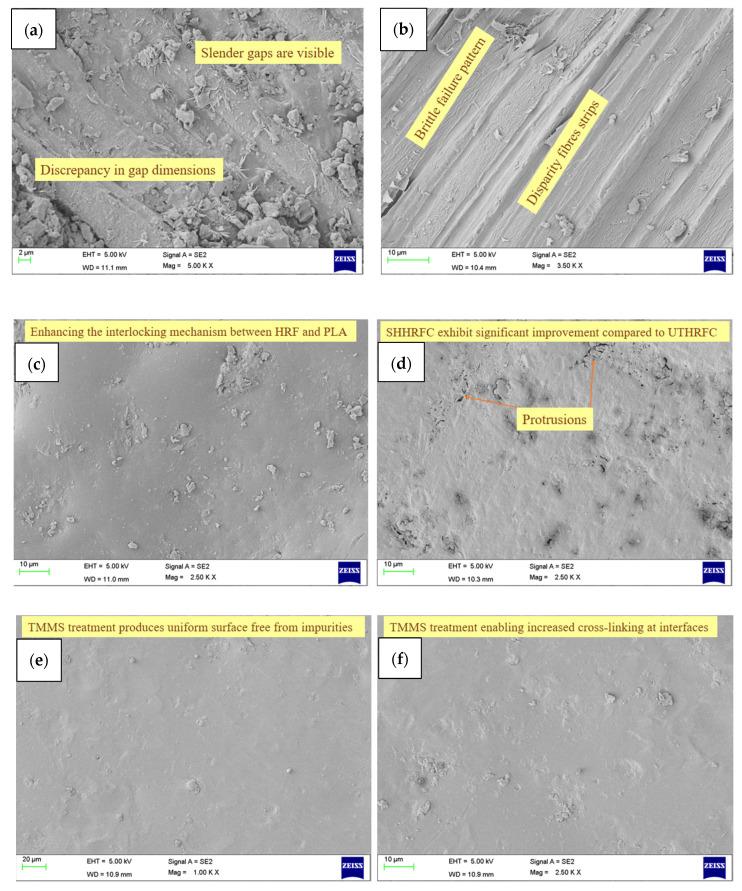
SEM images of (**a**,**b**) UHRFCs, (**c**,**d**) NHRFCs, and (**e**,**f**) THRFCs.

The structural features of UHRFCs are determined by the presence of important chemical elements such as lignin, cellulose, hemicelluloses, and wax. These constituents play an important role in improving the mechanical and thermal properties of HRFCs. Fractures within the matrix indicate a brittle failure mode, emphasizing an important element of the failure mechanism (Figure 12a,b). Upon examination of the images of the adhesion between the HRF and the PLA matrix, it can be seen that a gap exists between the fiber and the matrix. Minor gaps are observed in UHRFCs; however, larger gaps can be seen in the other samples. This mismatch in gap diameters greatly affects mechanical strength, indicating lower performance in UHRFCs.

The NaOH treatment has a two-fold effect on the surface properties of HRFs (Figure 12c,d). On the one hand, it causes roughness and increases protrusions, which improve the interlocking between HRF and PLA. This treatment causes visible fiber breakage, followed by the dissociation of linkages between the HRF and PLA, suggesting strong interfacial bonding. As a result, treated HRFCs have significantly better mechanical properties than UHRFCs. In contrast, NaOH treatment dissolves the wax and lignin content on the HRF surface, resulting in a loss in the mechanical strength of HRFCs. This decrease in mechanical strength, especially when compared to THRFCs, indicates a compromise in overall mechanical properties, despite the improved interfacial bonding attained with NaOH treatment.

The use of a coupling agent, such as TMMS, results in the development of a shielding layer around the HRFs (Figure 12e,f), resulting in significant improvements in thermal characteristics, as seen by the TGA results. Chemical treatments have had a positive impact on the morphology of HRF, providing a uniform surface without impurities. These treatments also create a protective layer on the fibers, contributing to increased thermal stability. The exact alignment of silane-treated HRFs with PLA yields significant improvements, with tensile, flexural, and toughness values of 118.62 MPa, 143.33 MPa, and 48.72 KJ/m^2^, respectively. The concentration of silane deposition increases, allowing for enhanced cross-linking at interfaces, thus strengthening the fiber-to-matrix adhesion and improving the overall mechanical characteristics.

## 4. Conclusions

The investigation into the properties of HRFCs fabricated by reinforcing fibers from *Hibiscus* plants for composite applications involved a comprehensive exploration of mechanical, visco-elastic, dielectric, and acoustic properties. From UHRFCs to NHRFCs, notable improvements are observed in mechanical properties, such as a 13.66% increase in tensile strength, a 15.98% increase in flexural strength, and a 4.19% enhancement in Shore-D hardness. Furthermore, the NHRFCs showed an increased dielectric constant by 23.52% and a remarkable 79.50% augmentation in sound absorption coefficient of 1897 Hz. Comparing UHRFCs, THRFCs demonstrated more substantial enhancements. THRFCs exhibited a remarkable 19.39% increase in tensile strength, a 33.07% increase in impact strength, and a 5% improvement in Shore-D hardness. Additionally, the dielectric constant increased by 35.29%, and the sound absorption coefficient of 1897 Hz saw a remarkable 81% improvement. THRFCs displayed a 2.90% rise in storage modulus and a 9.48% increase in sound absorption coefficient of 1897 Hz compared to NHRFCs. This result signifies that both NHRFCs and THRFCs exhibit substantial improvements in mechanical, dielectric, and acoustic properties compared to UHRFCs. Furthermore, the THRFCs show superior enhancements, suggesting the efficacy of TMMS treatment in enhancing diverse properties, potentially making it an excellent candidate for diverse industrial applications requiring superior performance and sustainability.

## Figures and Tables

**Table 1:** TGA results of PLA and HRFCs.

Parameters	PLA	UHRFCs	NHRFCs	THRFCs
Initial decomposition (°C)	97.45 ± 3.62	98.66 ± 4.13	98.13 ± 3.22	99.99 ± 2.11
Onset temperature (°C)	292.61 ± 4.15	304.15 ± 4.23	309.89 ± 3.63	312.99 ± 3.96
Peak temperature (°C)	353.86 ± 4.34	420.62 ± 3.43	439.51 ± 3.54	469.07 ± 3.11
Residue (%)	5.25 ± 0.09	7.44 ± 0.06	8.56 ± 0.03	9.71 ± 0.01
Total weight loss (%)	94.75 ± 0.87	92.56 ± 0.93	91.44 ± 0.67	90.29 ± 0.33
Activation energy (kJ/mol)	172. 82 ± 2.89	181. 26 ± 2.45	184. 31 ± 2.42	186. 75 ± 1.99

## Data Availability

The original contributions presented in this study are included in this article. Further inquiries can be directed to the corresponding author.
